# CAPABLE TRANSPLANT: ADAPTATION OF CAPABLE FOR USE WITH OLDER ADULTS WITH INACTIVE STATUS AWAITING KIDNEY TRANSPLANT

**DOI:** 10.1093/geroni/igae098.0585

**Published:** 2024-12-31

**Authors:** Melissa Hladek, Samantha Curriero, Qian-Li Xue, Deidra Crews, Mara McAdams DeMarco, Deborah Wilson, Daniel Brennan, Sarah Szanton

**Affiliations:** Johns Hopkins University, Baltimore, Maryland, United States; Johns Hopkins University, Baltimore, Maryland, United States; Johns Hopkins University, Baltimore, Maryland, United States; Johns Hopkins University, Baltimore, Maryland, United States; New York University, New York, New York, United States; Auckland University of Technology, Auckland, Auckland, New Zealand; Johns Hopkins University, Baltimore, Maryland, United States; Johns Hopkins University, Baltimore, Maryland, United States

## Abstract

Over 800,000 Americans live with end-stage kidney disease (ESKD), disproportionally affecting older adults. This group represents just 1% of Medicare’s Fee for Service (FFS) population, yet utilizes greater than 7% Medicare FFS resources. Rates of kidney transplantation (KT), the preferred treatment for ESKD, have risen 5-fold for older adults since 1990 but there is vast heterogeneity in access to KT. Older adults are more likely to be listed as inactive (on the waitlist but ineligible for KT) and, inactive status is associated with a 2.2 fold increased risk of waitlist mortality compared to those that remain active. Barriers to active status are multi-factorial but frequently modifiable and include symptoms such as fatigue, depression and stress, pain as well as loss of physical function, social isolation, and decreased health literacy. Using community participatory research and human centered design, we used photos, interviews, persona development, storyboarding and focus groups to identify three barriers unique to this population around the themes of: mental fortitude, support and communication. Our CAPABLE adaptation, called CAPABLE Transplant, incorporates greater motivational and communication strategies for the person and the home as well as a robust digital literacy component to address the needs of this population. An open-label pilot phase helped to solidify all aspects of the adaptation with resulting 30-person pilot randomized control trial on-going. This presentation will focus on the human centered design adaptation process and results thus far.

